# WEB-BASED COGNITIVE BEHAVIORAL THERAPY FOR DEPRESSION AMONG OLDER ADULTS: DEVELOPMENT AND USABILITY STUDY

**DOI:** 10.1093/geroni/igad104.2109

**Published:** 2023-12-21

**Authors:** Jay Kayser, Samson Ash, Chuxuan Zheng, Skyla Turner, Xiaoling Xiang

**Affiliations:** University of Michigan, Ann Arbor, Michigan, United States; University of Michigan, Ann Arbor, Michigan, United States; University of Michigan, Ann Arbor, Michigan, United States; University of Michigan, Ann Arbor, Michigan, United States; University of Michigan, Ann Arbor, Michigan, United States

## Abstract

Digital Mental Health Interventions (DMHIs) can potentially improve treatment access for older adults experiencing depression, but few tailored web-based interventions exist. This paper describes the design and development process of a web-based cognitive-behavioral therapy program called Empower@Home and reports outcomes from a usability evaluation. The program was developed in collaboration with community agencies, stakeholders, and older adults. Development was guided by user-centered design principles and informed by multiple data sources. A comparative usability evaluation with ten older adults was conducted to assess the usability of Empower@Home. Paired t-tests were used to compare scores on the System Usability Scale (SUS) between three interventions. In addition, field testing was conducted with 4 end-users to detect any further usability issues. The content and design of the intervention was heavily influenced by feedback and recommendations provided by our community partners. Empower@Home involves nine sessions, completed over ten weeks, and a printed workbook. The sessions involve psychoeducation, short video clips, interactive content, an animated storyline, and weekly out-of-session home practices. In the comparative usability evaluation, Empower@Home received statistically significantly higher usability scores than both comparison interventions, and 80% of users preferred Empower@Home over other interventions. In field testing, all participants “agreed” or “strongly agreed” that they liked the procedures used in Empower@Home and felt confident in performing program-related tasks. Empower@Home is highly responsive to end user needs and the implementation setting’s characteristics. Interventions that work with community stakeholders and thoughtfully consider implementation barriers in the design process will likely produce more usable and effective DMHIs.

